# Does earlier diagnosis and treatment of brain tumors matter? Time-to-treatment intervals and tumor size at detection; impact on survival and other patient outcomes

**DOI:** 10.1093/noajnl/vdag230

**Published:** 2026-09-28

**Authors:** Ewan Gray, James M Cameron, Abigail Lishman, Peter Hall, Piyumanga Karunaratne, Giovanni Tramonti, Maheva Vallet, Luke Pike, Matthew J Baker, Paul M Brennan

**Affiliations:** Freelance Consultant, Edinburgh, UK; Dxcover Ltd, Glasgow, UK; Dxcover Ltd, Glasgow, UK; Edinburgh Cancer Informatics, University of Edinburgh, Edinburgh, UK; Edinburgh Cancer Informatics, University of Edinburgh, Edinburgh, UK; Edinburgh Cancer Informatics, University of Edinburgh, Edinburgh, UK; Edinburgh Cancer Informatics, University of Edinburgh, Edinburgh, UK; Department of Radiation Oncology, Memorial Sloan Kettering Cancer Center, New York, USA; Dxcover Ltd, Glasgow, UK; School of Medicine & Dentistry, University of Lancashire, Preston, UK; Translational Neurosurgery, Centre for Clinical Brain Sciences, University of Edinburgh, Edinburgh, UK

**Keywords:** brain tumor, early diagnosis, tumor size

## Abstract

**Background:**

The importance of timely diagnosis of brain tumors is well recognized but how this translates to impact on important patient outcomes is not well described. This observational study aimed to quantify the effect of time-to-treatment interval and tumor size at diagnosis on six patient outcomes: tumor-specific survival, overall survival, new or worsened neurological deficit, 30-day mortality, recurrence, and inpatient length of stay.

**Methods:**

A retrospective cohort study including 1,196 patients from Southeast Scotland diagnosed with brain tumors between 2010 and 2020. Analysis was stratified by tumor type in three categories: Glioblastoma (GBM) and other grade 4 brain tumors, grade 1-3 tumors and brain metastases. Regression methods (Cox, Logistic & Linear) were used to estimate per day impact of time-to treatment and tumor size impact on outcomes.

**Results:**

The mean time from first presentation to treatment was 161 days (median: 57 days). Time-to-treatment results showed an association between longer intervals and reduced mortality. The mean tumor size was 4.06 cm (SD: 1.75cm). For GBM and grade 4 tumors, each 1cm increase in tumor size increased tumor-specific mortality risk by approximately 11% (HR: 1.11, 95%CI: 1.05-1.16) and increased all-cause mortality by 10% (HR: 1.10, 1.05-1.16). Associations between tumor size and the outcomes of brain tumor mortality, all-cause mortality and inpatient length of stay were consistent across tumor subgroups.

**Conclusions:**

Larger tumor size was consistently associated with increased hazard ratios for mortality. Applying these estimates together with estimates of mean tumor growth rates available in the literature, if a 6cm Glioblastoma tumor were diagnosed 3 months earlier, then we would expect a 17%-26% reduction in hazard of brain tumor mortality.

Key Points

- Larger tumor size was associated with increased mortality.
- Longer time-to-treatment interval was associated with reduced mortality, evidence of the “waiting time paradox.”

Importance of the Study

These findings reinforce the potential positive impact of earlier diagnosis for brain tumor patients. Building upon the accumulated evidence that tumor size is a critical factor influencing prognosis, this study is the first to focus on estimating causal associations with patient outcomes. Earlier detection, through influence on tumor size at diagnosis, may offer measurable survival benefits and potential reductions in healthcare resource use. There are limitations of an observational study design, but we believe our large, robust dataset and detailed analysis provide a stronger foundation for advocating further prospective research such as trials or pilots of early diagnostic interventions.

Diagnosis of brain tumors suffers from two related problems, an excess of emergency presentations^1^ and long diagnostic intervals.^2^ NHS England route-to-diagnosis data show that approximately 40% of primary brain cancers present as an emergency each year.^1^ In the United Kingdom, most (80%) brain tumor patients report one or more primary care visit in the months preceding diagnosis,^3^ and more than a third of patients have three or more primary care visits recorded.^4^ These data suggest opportunities for earlier diagnosis.^5^

Despite the potential for earlier diagnosis, interventions aiming to improve speed and accuracy of diagnosis face barriers in demonstrating their impact and value. While the importance of a timely diagnosis is recognized by professionals and policy makers, how this translates in a quantitative sense to important patient outcomes such as survival, quality of life and length of hospital stays, is not well described. Consequently, the impact of earlier diagnoses on population health and healthcare resource use are highly uncertain. This creates difficulty for the economic evaluation of interventions aiming to aid earlier brain tumor diagnosis.^6^^,^^7^ Evidence from real-world observational data can help address this.

The Edinburgh brain tumor database utilized by this study is well suited to interrogate the relationship between time to diagnosis and patient outcomes. This manually curated data was collected specifically to understand clinical pathways for brain tumor patients and can be supplemented through linkage to routine healthcare records.

Prior studies in this area have explored only limited aspects of the relationship between earlier (or delayed) diagnosis, treatment, and patient outcomes. The overall pattern of findings reveals inconsistency, with positive (longer times associated with improved outcome),^8^ negative,^9^^,^^10^ and null^11^ associations identified. An important part of the explanation of these seemingly contradictory findings is the “waiting time paradox”^8^^,^  ^12^^,^^13^- the phenomenon where a delayed diagnosis or treatment can paradoxically mean a better prognosis for the patient. This is due to differences between cases in clinical features that may induce delay and are also associated with better outcomes. For example, a longer diagnostic process is more likely for less severely impairing symptoms, and such symptoms may be more commonly associated with less aggressive disease.

To better elucidate the relationship between earlier or delayed diagnosis and patient outcomes, this study investigated two associations. First, the association between time-to-treatment and outcomes. Second, the association between tumor size and outcomes.

Tumor size is expected to mediate the impact of earlier or delayed diagnosis through the process of tumor growth. Pre-operative tumor size is a well-defined prognostic factor for both low- and high-grade brain tumors.^14-18^ Larger tumor size can increase the risk of both neurological deficits at presentation and surgical complications following resection.

While the relationship between tumor size and patient outcomes is well described in the literature, this study examines it from a unique perspective. Prior studies were concerned with the prognostic (predictive) value of tumor size rather than trying to estimate a causal effect.^19^ The multivariate analysis reported in these studies are inappropriate from a causal perspective due to including potential confounders such as tumor growth rate,^20^ mediators of the effect of tumor size such as extent of resection,^15^^,^^16^ or potential outcomes of tumor size (colliders) such as pre-operative symptoms.^16^^,^^17^ The reported results are therefore potentially biased estimates of the causal effect of tumor size on patient outcomes.

Patient outcome variables were selected based on known clinical relevance and availability in the database. The main objectives of the study were specified as estimation of the association between the two indicators of earlier or later diagnosis, (1) tumor size and (2) length of the presentation to treatment interval, with the patient outcomes of brain tumor specific survival and overall survival. Additional objectives were to estimate the association of these indicators with the patient outcomes of new or worsened neurological deficit, 30-day mortality, cancer recurrence and inpatient length of stay in 12 months following diagnosis.

## Methods

### Study Design

A retrospective cohort study.

### Patient Data

All patients were identified from the Edinburgh brain tumor database, which includes consecutive patients whose care was discussed at the institutional neuro-oncology multi-disciplinary meeting. Patients diagnosed with brain tumors between 1^st^ January 2010 to 31^st^ May 2020 were included in the study. Follow up was available until 31^st^ May 2021 for all patients.

Brain tumor diagnosis was defined as a radiological diagnosis with or without recorded pathology diagnosis. Brain tumor types were defined based on diagnosis labels manually recorded form the radiology report. Inclusion criteria limited the study to patients aged 18 and over. Exclusion criteria of previous history of other cancer within the last 3 years was applied.

### Data Processing

Patient information was collected from manually curated fields compiled in the database and supplemented by data-linkage with national routine datasets (SMR00—Outpatient, SMR01—Inpatient and SMR06—Cancer Registry). Pseudonymous data linkage was achieved by linking the patient’s database id number to their NHS Scotland Community Health Index (CHI) number. Data processing was completed within an NHS IT environment by an NHS analyst.

Tumor size was defined as the largest diameter of the tumor given in the diagnostic report (CT or MRI). Time-to-treatment interval was defined in two ways,^1^ as time from first presentation to first recorded treatment (any one of surgery [including biopsy], radiotherapy or Temozolomide). This is equivalent to the primary care interval plus the secondary care interval,^21^ and^2^ in sensitivity analysis only time from radiological diagnosis to first recorded treatment (the treatment interval only^21^). Time of first presentation was defined as the date of first recorded clinical appearance or the first date of recorded symptoms if this was earlier. Date of diagnosis refers to the date of the first imaging procedure that confirmed a radiological diagnosis. The date of treatment is the date first treatment was received, whether this was surgery, radiotherapy or chemotherapy.

Outcome variables included all-cause mortality, brain tumor specific mortality (Supplementary Appendix reports ICD-10 codes included for this outcome), 30-day (all-cause) mortality, new or worsened neurological deficit, tumor recurrence (confirmed radiologically), and total inpatient bed days within 12 months of the date of diagnosis. Brain tumor specific mortality and all-cause mortality were selected as the main outcomes of interest. While other outcomes were considered additional items to supplement these most clinically relevant outcomes.

Other demographic and clinical factors available for the descriptive analysis included age at diagnosis, sex, tumor location, tumor grade, route of presentation, Charlson comorbidity index, deprivation level (SIMD quintile), and urban/rural residence. Treatments received were recorded for the categories of surgery, radiotherapy, and use of temozolomide.

### Statistical Analysis

Analysis was stratified by tumor type. All procedures were repeated identically, limited to patients in each of three brain tumor categories. Tumors were grouped as Glioblastoma (GBM) and other grade 4 primary brain tumors (Grade 4 group), Grade 1-3 primary brain tumors (Grade 1-3 group), and Brain metastases. Results were also produced for the pooled sample including all three groups. The categories were chosen based on clinical relevance while still being sufficiently broad to allow meaningful results. This was conducted with the intention explore the consistency of effects across these groups. Note that the metastases group included in the database was intentionally limited to exclude those with known active primary tumors at other sites (i.e., it includes those that presented with a brain tumor and subsequently discovered to be secondary to another site).

Mortality outcomes analyzed by survival analysis were considered the primary outcomes. Hazard ratios were estimated for cancer-specific, overall mortality and tumor recurrence using Cox regression, including covariates to adjust estimates for potential confounding variables. For the additional outcomes of new or worsened neurological deficit, and 30-day mortality, odds ratios were estimated by logistic regression. Linear regression was used to estimate effects on the number of inpatient bed days. This was subject to first identifying if a linear relationship offered a reasonable approximation based on examining scatterplots and QQ plots.

The covariates included in all Cox, Logistic, or Linear regressions were age at diagnosis, sex, deprivation level (SIMD quintile), Charlson comorbidity index, and Glioblastoma (binary indicator, only applies for the pooled cohort). These variables were assessed to be potential confounders to the relationship between the exposures and outcomes. Clinical variables that we assessed to be mediators, downstream of time-to-treatment and tumor size, were excluded. This included features of presentation such as symptoms, as well as treatment intention and treatments received. Further potential confounders, such as molecular marker status were considered but not included to limitations to data and sample size.

After examining the time-to-treatment data distributions and associations it was noted that interpretation was made challenging due to the presence of outliers. This caused estimated coefficients to be extremely small and in many cases indistinguishable from zero up to the fourth decimal place. To address this, a post-hoc analysis which stratified on time-to-treatment, using clinically relevant categories was added. The categories applied were <2 weeks, 2-6 weeks, 6-12 weeks, and >12 weeks.

Complete case analysis was conducted therefore patients with missing data in relation to predictor, covariate, or outcomes variables were excluded from the regression analysis.

## Results

Of the 1437 patients, 1196 entered in the database were included in the study (Figure 1), comprising of 552 patients with Glioblastoma and other grade 4 tumors, 494 with grade 1-3 primary brain tumors and 150 with brain metastases. Descriptive statistics are displayed in Table 1. The mean age was 63. The mean tumor size was 40.6mm (SD: 17.5mm). Tumors were most commonly located in the frontal lobe (36.3%). Tumor size data was missing for 126 patients.

**Figure 1.**
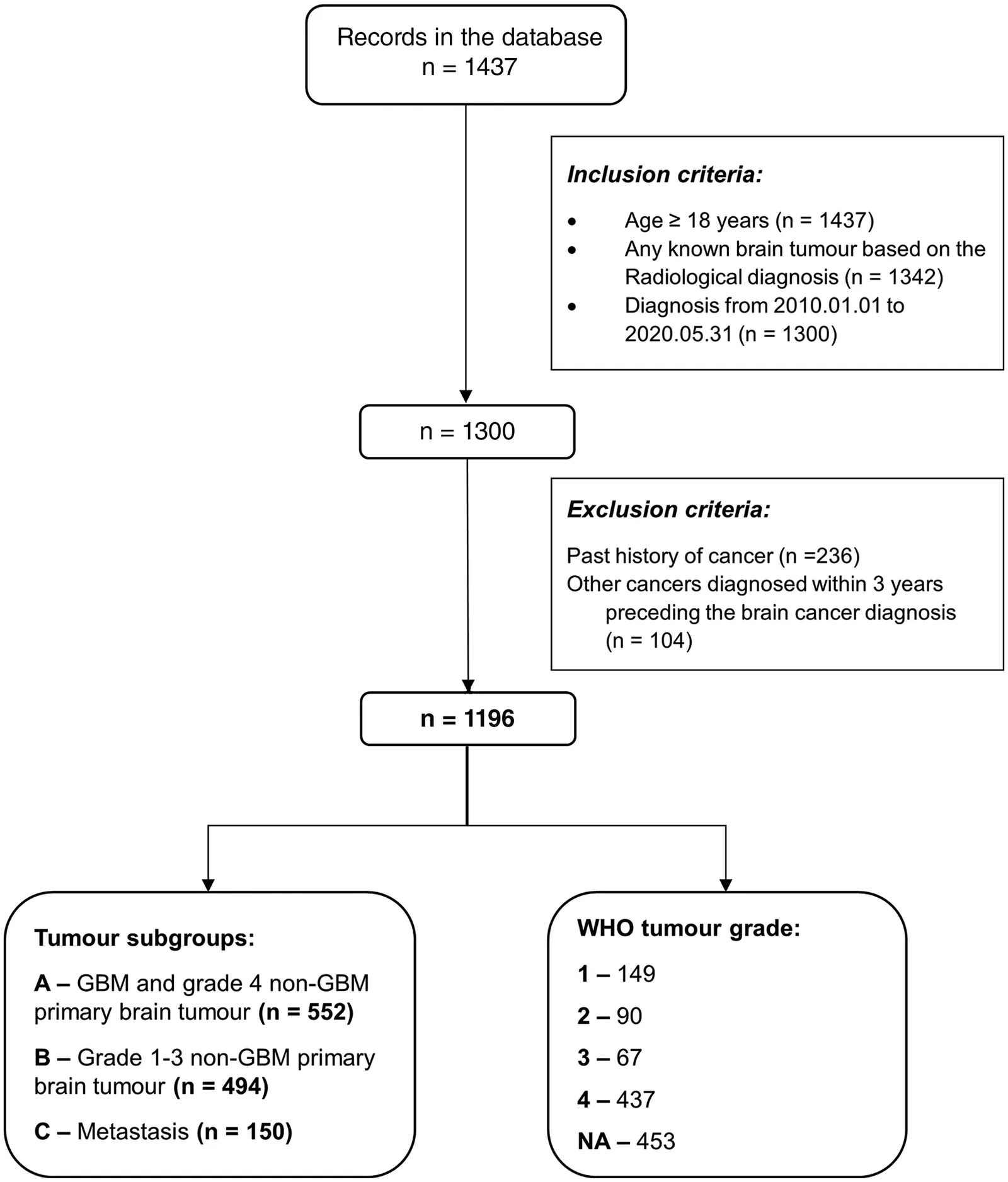
Participant flow, subgroups and exclusions.

**Table 1.** Descriptive statistics

	GBM + Grade 4 non-GBM primary tumor (N = 552)	Grade 1-3 non-GBM primary tumor (N = 494)	Brain metastases (n = 150)	All (n = 1196)
**Age at diagnosis**				
Mean (SD)	62.0 (13.2)	55.2 (17.2)	64.0 (11.4)	59.4 (15.2)
Median (Q1, Q3)	64.0 (54.0, 71.0)	54.0 (43.0, 69.0)	65.0 (56.2, 72.0)	61.0 (50.0, 70.0)
**Sex**				
Female	226 (40.9%)	275 (55.7%)	75 (50.0%)	576 (48.2%)
Male	326 (59.1%)	219 (44.3%)	75 (50.0%)	620 (51.8%)
**Primary cancer subtype**				
Glioblastoma	459 (83.2%)	0 (0.0%)	0 (0.0%)	459 (38.4%)
Other Glioma	83 (15.0%)	210 (42.5%)	0 (0.0%)	293 (24.5%)
Meningioma	<10 (<1.8%)	>168 (>34%)	0 (0.0%)	178 (14.9%)
Other primary brain cancer	<10 (<1.8%)	>98 (>20%)	0 (0.0%)	116 (9.7%)
Metastasis	0 (0.0%)	0 (0.0%)	150 (100.0%)	150 (12.5%)
**Tumor size at diagnosis (cm)^a^**				
Mean (SD)	4.6 (1.7)	3.8 (1.8)	3.0 (1.2)	4.1 (1.8)
Median (Q1, Q3)	4.5 (3.4, 5.7)	3.7 (2.4, 5.0)	3.0 (2.2, 3.7)	4.0 (2.8, 5.3)
Number Missing	>40	>75	<10	126
**Tumor size category (cm)**				
<3	81 (15.9%)	143 (<40%)	69 (<48%)	293 (27.4%)
3-5.9	315 (61.8%)	213 (≥50%)	73 (≥50%)	601 (56.2%)
≥6	114 (22.4%)	>50 (>10%)	<10 (<2%)	176 (16.4%)
Number Missing	>40	>75	<10	126
**Tumor location**				
Frontal	196 (36.8%)	161 (37.5%)	46 (30.9%)	403 (36.3%)
Temporal	125 (23.5%)	74 (17.2%)	11 (7.4%)	210 (18.9%)
Parietal	88 (16.5%)	56 (13.1%)	20 (13.4%)	164 (14.8%)
Occipital	≥10 (≥1.8%)	≥10 (≥2%)	<10 (<6.6%)	36 (3.2%)
Cerebellar	<10 (<1.8%)	≥10 (≥2%)	33 (22.1%)	64 (5.8%)
Brainstem	<10 (<1.8%)	33 (7.7%)	<10 (<6.6%)	37 (3.3%)
Multiple	103 (19.3%)	62 (14.5%)	32 (21.5%)	197 (17.7%)
Number Missing	≥10	65	<10	85
**Tumor grade**				
1	<10 (<1.9%)	143 (37.1%)	<10 (<20%)	149 (20.1%)
2	<10 (<1.9%)	87 (22.6%)	<10 (<20%)	90 (12.1%)
3	<10 (<1.9%)	62 (16.1%)	<10 (<20%)	67 (9.0%)
4	413 (98.1%)	0 (0.0%)	24 (80.0%)	437 (58.8%)
Number Missing	131	202	120	453
**Presentation**				
Primary care	22 (6.2%)	40 (10.3%)	14 (11.1%)	76 (8.7%)
Secondary care	182 (51.3%)	224 (57.4%)	61 (48.4%)	467 (53.6%)
Emergency	151 (42.5%)	126 (32.3%)	51 (40.5%)	328 (37.7%)
Number Missing	197	104	24	325
**Treatment received (Surgery, Radiotherapy and/or Temozolomide)**				
Yes	446 (80.8%)	362 (73.3%)	103 (68.7%)	911 (76.2%)
No	106 (19.2%)	132 (26.7%)	47 (31.3%)	285 (23.8%)
**Surgery**				
Yes	425 (77.0%)	346 (70.0%)	87 (58.0%)	858 (71.7%)
No	127 (23.0%)	148 (30.0%)	63 (42.0%)	338 (28.3%)
**Radiotherapy**				
Yes	390 (70.7%)	188 (38.1%)	66 (44.0%)	644 (53.8%)
No	162 (29.3%)	306 (61.9%)	84 (56.0%)	552 (46.2%)
**Temozolomide**				
Yes	249 (45.1%)	64 (13.0%)	17 (11.3%)	330 (27.6%)
No	303 (54.9%)	430 (87.0%)	133 (88.7%)	866 (72.4%)
Comorbidity (CCI)*				
0	477 (86.4%)	436 (88.3%)	96 (64.0%)	1009 (84.4%)
1-2	59 (10.7%)	46 (9.3%)	18 (12.0%)	123 (10.3%)
3 or more	16 (2.9%)	12 (2.4%)	36 (24.0%)	64 (5.4%)
Deprivation score (SIMD)**				
1 (Most deprived)	>60 (>10%)	<70 (<14%)	<20 (<13%)	>140 (>11%)
2	125 (22.7%)	110 (22.5%)	29 (19.5%)	264 (22.2%)
3	>100 (>18%)	<100 (<20%)	<40 (<27%)	>240 (>20%)
4	130 (23.6%)	107 (21.9%)	28 (18.8%)	265 (22.3%)
5 (Least deprived)	124 (22.5%)	114 (23.3%)	35 (23.5%)	273 (23.0%)
Number Missing	<10	<10	<10	<10
**Urban/Rural residence**				
Urban	399 (76.7%)	347 (82.0%)	108 (80.0%)	854 (79.2%)
Rural	121 (23.3%)	76 (18.0%)	27 (20.0%)	224 (20.8%)
Number Missing	32	71	15	118
**Healthcare Resource Use**				
**Outpatient visits**				
Mean (SD)	4.6 (3.3)	5.2 (3.7)	6.1 (5.0)	5.0 (3.7)
Median (Q1, Q3)	4.0 (2.0, 6.0)	5.0 (2.0, 7.0)	5.0 (2.0, 9.0)	4.0 (2.0, 7.0)
Number Missing	84	48	23	155
**Inpatient visits**				
Mean (SD)	5.3 (3.1)	4.8 (3.1)	6.0 (3.8)	5.2 (3.2)
Median (Q1, Q3)	5.0 (3.0, 7.0)	4.0 (3.0, 6.0)	5.0 (3.0, 7.0)	4.0 (3.0, 7.0)
Number Missing	33	101	11	145
**Total length of stay (days)**				
Mean (SD)	31.8 (32.8)	28.3 (39.4)	34.5 (32.4)	30.8 (35.4)
Median (Q1, Q3)	20.0 (7.5, 47.0)	13.0 (6.0, 36.0)	24.0 (13.0, 48.0)	18.0 (7.0, 44.0)
Number Missing	33	101	11	145
**Average length of stay (days)**				
Mean (SD)	6.3 (6.3)	5.7 (6.6)	6.7 (7.2)	6.1 (6.5)
Median (Q1, Q3)	4.1 (2.0, 8.7)	3.0 (1.8, 7.2)	4.7 (2.4, 7.5)	3.8 (2.0, 7.9)
Number Missing	33	101	11	145
**Time from radiological diagnosis to first treatment (days)**				
Mean (SD)	30.0 (75.2)	173.5 (395.3)	41.5 (123.8)	88.3 (267.0)
Median (Q1, Q3)	17.0 (10.0, 27.0)	43.5 (15.0, 126.8)	19.0 (12.0, 36.0)	21.0 (12.0, 49.0)
Number Missing	106	132	47	285
**Time from first presentation to treatment (days)**				
Mean (SD)	75.0 (135.6)	297.0 (469.6)	73.5 (133.2)	161.5 (329.9)
Median (Q1, Q3)	41.5 (25.0, 77.8)	116.0 (48.8, 338.8)	41.0 (27.0, 70.0)	57.0 (30.0, 132.5)
Number Missing	118	154	53	325
**Time from primary care referral to treatment (days)**				
Mean (SD)	37.0 (60.5)	202.2 (362.4)	35.4 (44.6)	105.1 (250.4)
Median (Q1, Q3)	19.0 (11.0, 40.5)	65.0 (21.8, 205.2)	22.0 (14.0, 41.2)	28.0 (13.5, 82.5)
Number Missing	257	230	70	557

Small numbers are censored due to information governance requirements of the source data.

a The assessments of tumor size recorded were based on standard care as reported by consultant radiologist. For tumors with contrast enhancement, including all GBM, Meningioma and metastases the measurement is from the T1-weighted post gadolinium imaging, including the maximum area confined by the peripheral contrast enhancement. For low grade gliomas it is T2-weighted hyperintense region.

The most common route to diagnosis was via the emergency department (37.7%) and the majority of patients received surgical treatment (72%). In addition, 54% received radiotherapy treatment and 28% recorded use of temozolomide.

Survival varied by tumor type in the expected pattern. Median survival was 9 months for the Grade 4 group, 123.6 months for Grade 1-3 group and 7.6 months for metastases (Figure 2).

**Figure 2.**
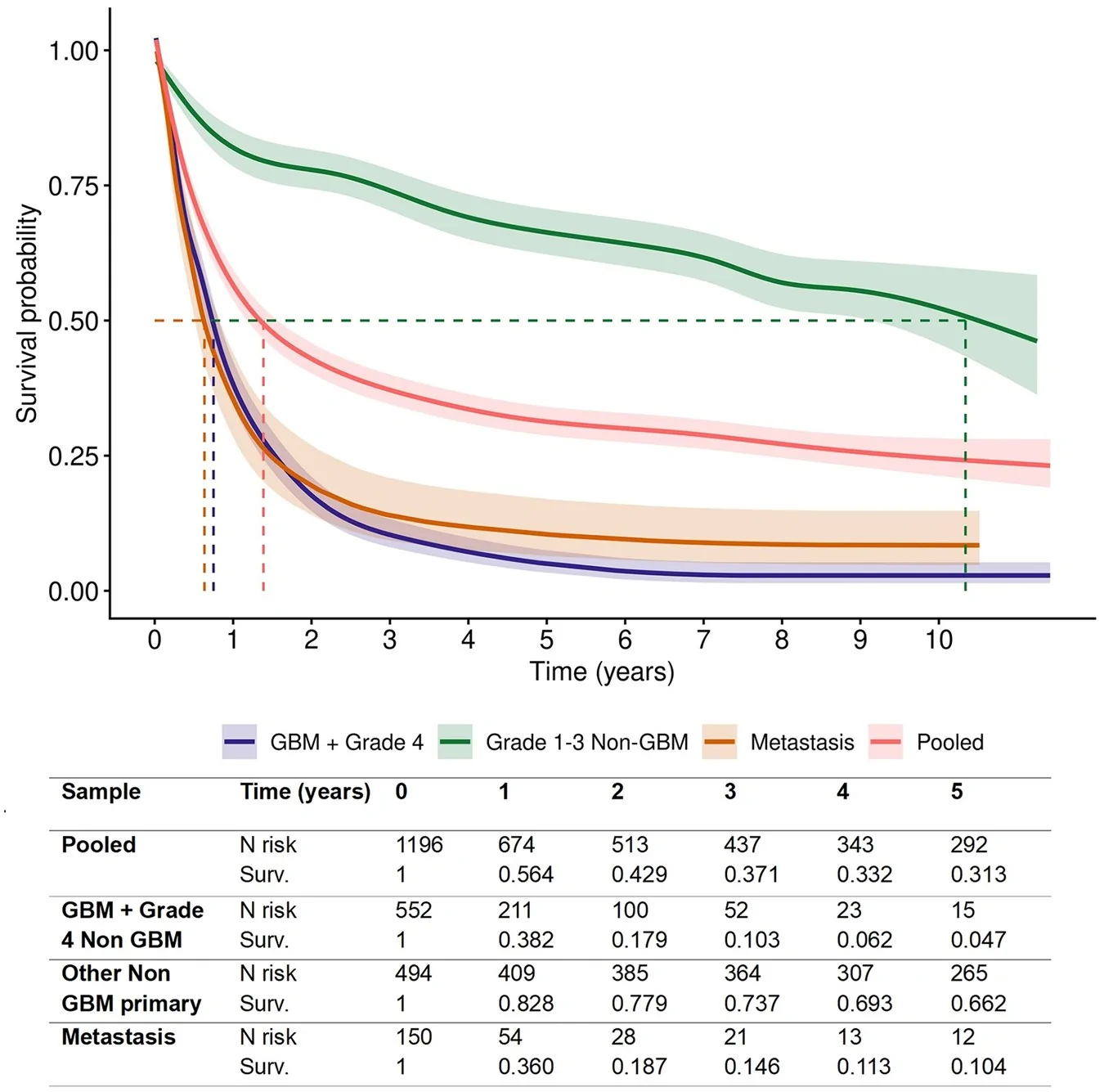
Kaplan-Meier survival analysis of overall survival, tumor type subgroups and pooled cohort. Patients still alive at the latest passive follow-up were right-censored. Numbers at risk displayed below.

The time in clinical pathways patients followed from first presentation through referrals, diagnosis, and initial treatment are summarized in Table 1. The mean time from first presentation to treatment in the pooled sample was 161 days (median: 57 days) and was missing in 324 patients. Time interval varied by tumor type, for Grade 4 patients the mean was 75 days (median: 42 days), for non-GBM PBT patients it was 297 days (median: 116 days) and for patients with metastases 73 days (median: 40 days). Means being higher than medians, and large standard deviations (136-470 days), indicate strongly right skewed distributions in all categories. The difference in means between presentation to treatment and diagnosis to treatment indicates an average of around 40 days from presentation to diagnosis, with wide variation. High variance in these intervals reflects both variation in the duration until symptoms are recognized as a possible tumor, and variation in triage following diagnosis. For patients some with grade 1-3 primary brain tumors the variance is likely to also reflect watchful waiting strategies following diagnosis.

Associations between tumor size and patient outcomes are reported in Table 2. Tumor size (per cm larger) was associated with an increased risk of mortality from brain tumor. The direction of the association was observed to be consistent across tumor types (Figure S1 and Table 2). The hazard ratio for tumor-specific mortality was 1.11 (95%CI: 1.05-1.16) for grade 4 tumors, 1.24 (95%CI: 1.12-1.37) for grade 1-3 tumors and 1.15 (95%CI: 0.96-1.39) for metastases. Equivalent hazard ratios for all-cause mortality were 1.10 (1.05-1.16) for grade 4 tumors, 1.15 (1.05-1.25) for grade 1-3 tumors and 1.21 (1.02-1.42) for metastases. Associations in the pooled cohort are reported in the Table S1.

**Table 2.** Tumor size and patient outcomes association (multivariable adjusted)

Main Outcomes	GBM + Grade 4 non-GBM primary tumor (N = 508)	Grade 1-3 non-GBM primary tumor (N = 413)	Brain metastases (n = 143)
Hazard Ratio, Odds Ratio, Coefficient (95% Confidence Interval)			
Brain cancer mortality	1.11 (1.05-1.16)	1.24 (1.12-1.37)	1.15 (0.96-1.39)
All-cause mortality	1.10 (1.05-1.16)	1.15 (1.05-1.25)	1.21 (1.02-1.42)
Additional outcomes			
30-day mortality	1.50 (1.09-2.08)	0.87 (0.53-1.32)	1.46 (0.83-2.70)
Recurrence	0.91 (0.84-0.98)	1.26 (1.14-1.40)	1.05 (0.81-1.35)
New or worsened neurological deficit	1.04 (0.92-1.17)	1.27 (1.10-1.48)	0.89 (0.56-1.38)
Inpatient bed days, 12 months following diagnosis*	1.31 (−0.45-3.08) [Obs. = 480]	2.20 (−0.01-4.40) [Obs. = 332]	1.09 (−3.68-5.86) [Obs. = 132]

* Reduced number of observations due to missing outcome data, number of observations given in square brackets.

An association with increased risk of 30-day mortality was observed but this was only significant in the grade 4 group (OR: 1.50 95%CI: 1.09-2.08).

In total 445 recurrence events were observed (37% of the sample). There was evidence of reduced rate of recurrence in the grade 4 group (HR: 0.91, 95%CI: 0.84-0.98), and an increased rate of recurrence with increasing tumor size for grade 1-3 tumors (HR: 1.26, 95%CI: 1.14-1.40).

New or worsened neurological deficit and longer total duration of inpatient stays were also associated with larger tumor sizes in some groups (Table 2). Compared to tumor-specific and all-cause mortality more variation and wider confidence intervals were observed across tumor types in relation to these outcomes. For grade 1-3 tumors larger size was associated with increased risk of new or worsened neurological deficit (OR: 1.27, 95%CI: 1.10-1.48) but this was not observed for grade 4 tumors (OR: 1.04, 95%CI: 0.92-1.17) or brain metastases (OR: 0.89, 95%CI: 0.56-1.38).

Equivalent unadjusted (univariate) results for all associations of tumor size with outcomes, both in the pooled sample and stratified by tumor type, are available in the Supplementary Appendix (Tables S2-S5). Unadjusted results are very similar to the presented regression adjusted estimates with some small attenuation of effects following adjustment observed.

In the grade 1-3 group, increased time-to-treatment (per day, from first presentation) was associated with improved outcomes (Table 3) in relation to brain tumor mortality (HR: 0.9995, 95%CI: 0.9988-1.0001), all-cause mortality (HR: 0.9987, 95%CI: 0.9980-0.9995), and tumor recurrence (HR : 0.9996, 95%CI : 0.9992-1.0000). It should be noted that the combination of using hazard ratios for a per day change, and existence of long intervals, leads to very small coefficient values, making interpretation of clinical importance challenging. To illustrate the potential clinical relevance, the per day hazard ratio of brain tumor mortality of 0.9995 in the translates to a hazard ratio of 0.91 for a 180-day longer interval.

**Table 3.** Time from first presentation to treatment and patient outcomes association

Main Outcomes	GBM + Grade 4 non-GBM primary tumor (N = 432)	Grade 1-3 non-GBM primary tumor (N = 338)	Brain metastases (N = 96)
Hazard Ratio, Odds Ratio, Coefficient (95% Confidence Interval)			
Brain cancer mortality	0.9996 (0.9989-1.0004)	0.9995 (0.9988-1.0001)	0.9973 (0.9920-1.0026)
All-cause mortality	0.9998 (0.9991-1.0005)	0.9987 (0.9980-0.9995)	0.9993 (0.9975-1.0012)
Additional Outcomes			
30-day mortality	0.8671 (0.5168-1.0029)	1.0000 (0-3.075 x 10^35^)	0.8951 (0.4792-1.0162)
Recurrence	0.9996 (0.9985-1.0007)	0.9996 (0.9992-1.0000)	0.9979 (0.9934-1.0023)
New or worsened neurological deficit	0.9994 (0.9973-1.0009)	1.0001 (0.9996-1.0006)	0.9993 (0.9921-1.0031)
Inpatient bed days, 12 months following diagnosis	n/a	n/a	n/a

In the grade 4 and brain metastases groups, no clear association between time-to-treatment and better or worse patient outcomes was identified for any of the outcomes of interest.

The Supplementary Appendix provides results for unadjusted regressions (Tables S6, S8, S10, and S12), results for the alternative time-to-treatment definition (Tables S6-S13), and results for the same analyses stratified by categories of time-to-treatment window (Tables S14-S21). This sensitivity analysis found decreased risk of mortality with increased time-to-treatment in the 2- to 6-week and 6- to 12-week windows. There was little evidence of any association among those with greater than 12 weeks or less than 2 weeks duration of time-to-treatment.

Analysis stratified by time-to-treatment window categories (<2 weeks, 2-6 weeks, 6-12 weeks, and >12 weeks) shows similar results to the base case analysis, improved outcomes with longer time windows, but with larger effect sizes observed in the 2- to 6- and 6- to 12-week groups (Tables S14-S21).

Linear regression to estimate the relationship between time-to-treatment and inpatient length of stay was not applied because visual inspection of scatterplot and QQ plot did not suggest any approximately linear relationship was present.

## Discussion

Causal effects of earlier or later diagnosis of brain tumors are difficult to identify because experimental methods cannot be used, and observational data are challenging to collect and interpret. To overcome this difficulty, we have investigated the relationship between tumor size and patient outcomes as it is believed to play an important role mediating the effect of earlier or delayed diagnosis. This approach is predicated on a simple chain of causation. Tumors grow over time, and for brain tumors, size of the primary tumor has a very direct relationship with the severity of disease as well as the likelihood of successful treatment. Larger tumors will affect healthy brain tissues to a greater degree, compromising function, while complete and partial resections will carry greater risks of surgical complications. Delays to diagnosis allow greater time for tumor growth.

A key limitation of this study comes from the need to create broad categories of tumor types; GBM and other grade 4 PBT, grade 1-3 PBT and brain metastases. The grade 1-3 PBT category in particular includes diverse tumor types with highly heterogenous management. The grouping was clinically informed and reflects the grading system in use. We hypothesized that the relationship between changing tumor size and outcomes was nevertheless broadly similar across tumor types. Future studies should consider further dividing tumor subtypes into clinically relevant categories to the extent the data allows.

We have found evidence of the previously described “waiting time paradox” in the relationship between time-to-treatment and patient outcomes for grade 1-3 primary tumors. In common with previous investigators, we believe this is due to differences between cases in observed and unobserved prognostic factors. Prognostic factors affect how clinicians and patients assess the urgency of investigation and treatment. For example, in patients with similar symptoms, those whose symptoms progress more rapidly are likely to have diagnostic imaging earlier. Tumor growth and symptom progression is more likely with more aggressive tumors, which have a worse prognosis than lower grade tumors. Lower grade tumors grow more slowly, so symptoms will progress at a lower rate and diagnosis may therefore take longer, but these tumors have a better outcome. Hence, why for some patients a longer time to diagnosis is associated with a better outcome. This will also be impacted by greater use of observation in management of patients with better prognosis. Future studies could consider structured data collection of triage decisions and use of planed observation to better inform the effect of time-to-treatment.

We have observed several features that should increase our confidence in a causal interpretation of the impacts of tumor size on the patient outcomes of survival, brain tumor specific or all-cause, and inpatient length of stay. These include a strong association, clear temporality, a gradient of effect, consistency, and clinical plausibility.^22^ First, a strong association was observed in relation to cancer-specific mortality, all-cause mortality, and length of inpatient stay. Temporality is clear as tumor size is recorded at the time of radiological diagnosis. The expected gradient was observed in relation to length of inpatient stay. The observed associations were consistent across tumor types in relation to cancer-specific and all-cause survival and were consistent for inpatient stays except for brain metastases. Finally, because the mainstay of treatment is surgical resection and, ceteris paribus, surgery becomes more difficult (and urgent) with increased tumor size, the case for clinical plausibility is strong.

To illustrate the potential importance of the relationship between time-to-diagnosis, tumor size and outcomes consider as an example a patient with a GBM 60mm diameter at diagnosis (75th percentile in this patient population). Applying two alternative mean growth rate estimates derived by previous studies,^23^^,^^24^ this tumor would be expected to be 30.9-42.5 mm if diagnosed 3 months earlier. The reduction in tumor size would imply a 17%-26% decrease in the hazard of brain cancer mortality. In this example that would translate to approximately 37%-42% rather than 30% of patients surviving at 1 year, and 5%-7% rather than 3% of patients surviving at 3 years following diagnosis. Inpatient bed days would be reduced from approximately 34.7 to 30.8-32.4 days, saving approximately £1,150-2,000 in healthcare expenditure^25^ (details of calculations for this worked example are provided in the Supplementary Appendix).

The impact of such earlier detection for the whole population of brain tumor patients, across tumor types and size at diagnosis, could be predicted in a future modeling study, providing input for additional analysis of the overall impact of early diagnosis. This would support economic evaluation of interventions targeting earlier diagnosis. In addition, future modeling can inform the impacts for even greater reductions in the time to diagnosis, for example from potential screening interventions.

An interesting further conclusion arising from this model of tumor growth and early detection is that because tumors grow approximately exponentially, the period of the last doubling in size prior to detection via symptoms is vitally important. This is when each day or week of earlier detection will have the largest effect on the tumor size at diagnosis. It follows that a few weeks can make a difference in the symptomatic setting while by the same logic it may also be true that a few weeks would be inconsequential in the setting of asymptomatic screening.

It is important to note the limitations imposed by the observational design of this study. Without having recourse to experimental data on delays to diagnosis and treatment, which could only become available through a natural experiment, uncertainty will remain about whether the observed associations are causal. More definitive evidence may require interventional studies aimed at early detection with new technologies by either modification of diagnostic pathways or by screening. These studies would be necessarily large, expensive, and lengthy. Further observational studies may be able to provide larger samples for more detailed subgroup analysis, or allow inclusion of additional covariates such as brain edema and other clinical features that are potential confounders for the relationship between tumor size and outcomes.

Another limitation of this study is possible error in assigning the correct time of diagnosis or first presentation based on review of health care records. The true first presentation may not have been recognized or recorded within primary care. While we believe this does not have a major impact in these data if it were the case then this would have the effect of artificially shortening the observed intervals. Around 10% of cases were excluded in the tumor size analysis due to missing data. We believe this in unlikely to cause substantial bias as the proportion is small and we do not believe missingness is related to the outcomes of interest. However, we cannot exclude the possibility.

Here, we have utilized a large brain tumor database linked to routine healthcare data to investigate the potential of earlier or delayed diagnosis to impact six important patient outcomes. Provided that we accept the logic that relates tumor size to earlier or later diagnosis, this study finds consistent evidence that delay does matter for brain tumor patients. New interventions leading to earlier diagnosis can hope to improve patient outcomes. Further studies, including prospective trials or real-world piloting, may be needed to confirm the impact of any specific early diagnosis intervention.

## Supplementary Material

vdag230_Supplementary_Data

## Data Availability

Under data governance rules these patient data can be accessed by researchers via application to Public Health Scotland (PHS) electronic Data Research and Innovation Service (eDRIS).
