# Supplementary material for: Does earlier diagnosis and treatment of brain tumors matter? Time-to-treatment intervals and tumor size at detection; impact on survival and other patient outcomes
Source: Neurooncol Adv. 2026 Sep 28;8(1):vdag230. doi: 10.1093/noajnl/vdag230 (PMC13671752; doi:10.1093/noajnl/vdag230)
Supplement: Supplementary file 1 [file vdag230_supplementary_data.docx]

Supplementary Appendix

Contents

[1. Further information about variables included in the analysis	1](#_Toc206763188)

[2. Details of calculation of earlier detection example	1](#_Toc206763189)

[3. Tumour size and time-to-treatment associations in pooled cohort	3](#_Toc206763190)

[4. Tumour size associations with patient outcomes (unadjusted)	4](#_Toc206763191)

[5. Time-to-treatment associations with patient outcomes (continuous specification, unadjusted and adjusted)	6](#_Toc206763192)

[6. Time-to-treatment associations with patient outcomes, stratified by time-to-treatment category (continuous specification, unadjusted and adjusted)	10](#_Toc206763193)

[7. Number of Observations in Regression Analysis	15](#_Toc206763194)

[8.Supplementary Figures	17](#_Toc206763195)

## 1. Further information about variables included in the analysis

Brain tumour specific mortality definition: Cause of death matches any of these ICD-10 diagnoses: C700, C701, C709, C710, C711, C712, C713, C714, C715, C716, C717, C718, C719, C720, C721, C722,C723, C724, C725, C728, C729, C752, C753, C793, D320, D321, D329, D330, D331, D332, D333, D334, D339, D352, D354, D420, D429, D430, D431, D432, D433, D434, D444, D445.

## 2. Details of calculation of earlier detection example

A reduction in tumour size ($S_{r}$) due to earlier detection can be calculated from the size at detection ($S_{d}$) and an assumed exponential growth rate ($r$):

$$S_{e}=S_{d} \times\left( \frac{1}{1+r^{t}} \right)$$

$$S_{r}=S_{e}-S_{d}$$

$S_{e}$ is the size at earlier detection and $t$ is time in days.

The average volumetric growth rate for GBM tumours has been estimated to be 1.2% (Swanton et al) and 2.2% (Feucht et al) per day, based on reported tumour volume or size doubling times. These two growth rate estimates, converted to growth rates for the maximum diameter assuming a spherical shape, were used in the example calculation.

The impact of an $S_{r}$ magnitude reduction in size on outcomes was calculated by applying the estimated OR/HR from regression analysis. reduction. For example, for outcomes such as cancer specific mortality with an estimated hazard ratio:

$${HR}_{Sr}= e^{\log(HR)S_{r}}$$

In the example provided, a 60mm tumour with an average growth detected three months earlier would be expected to be between 17.5mm and 29.1mm smaller at that time. These reductions in size translate to hazard ratios of 0.83 and 0.74, equivalent to relative risk reductions of 17% and 26% respectively.

Relative risk reductions were translated to survival at 1 year and 3 years by assuming an exponential distribution of survival times, and survival for a tumour size of mean size (40mm) being approximately equal to mean survival.

The linear regression coefficients estimated for inpatient bed days were applied directly to the size reductions above. Cost savings were calculated using an inpatient bed day cost for a neurosurgical unit of £500 (Scottish Costs book).

## 3. Tumour size and time-to-treatment associations in pooled cohort

SA Table 1 Tumour size and time-to-treatment associations for main outcomes and additional outcomes in the pooled cohort

|  | Tumour size |  | Time-to-treatment |
| --- | --- | --- | --- |
| Main Outcomes | Pooled cohort  (n = 1064) |  | Pooled cohort  (n = 866) |
|  | ***Hazard Ratio, Odds Ratio, Coefficient (95% Confidence Interval)*** | | |
| Brain cancer mortality | 1.11  (1.06 – 1.16) |  | 0.9993  (0.9988 - 0.9998) |
| All-cause mortality | 1.06  (1.02 – 1.11) |  | 0.9988  (0.9982 - 0.9993) |
| Additional Outcomes |  |  |  |
| 30-day mortality | 1.19  (0.96 – 1.49) |  | 0.8814  (0.6935 - 0.9816) |
| Recurrence | 1.00  (0.95 – 1.06) |  | 0.9995  (0.9991 - 0.9999) |
| New or worsened neurological deficit | 1.11  (1.02 – 1.22) |  | 1.0001  (0.9996 -1.0006) |
| Inpatient bed days, 12 months following diagnosis* | 1.52  (0.23 - 2.81)  [Obs. = 944] |  | n/a |

## 4. Tumour size associations with patient outcomes (unadjusted)

Note the number of observations included in each analysis varies depending on the necessary variables and levels of missingness. The number of observation for each are reported in SA Table 21 to SA Table 23.

SA Table 2: Association between tumour size and outcomes of interest (unadjusted) - overall

|  | **Outcome** | | | | | |
| --- | --- | --- | --- | --- | --- | --- |
|  | **30-day mortality**  (n = 1070) | | **New or worsened neurological deficit**  (n = 1070) | | **Inpatient bed days up to 12 months following diagnosis** (n = 949) | |
| **Predictor** | *Unadjusted OR*  *(95% CI)* | *p-value* | *Unadjusted OR*  *(95% CI)* | *p-value* | *Unadjusted Coefficient (SE)* | *p-value* |
| Tumour size (cm) | 1.21  (0.99 – 1.47) | 0.063 | 1.16  (1.07 – 1.27) | **<0.001*** | 1.50 (0.64) | **0.020*** |
| **Cont.** | | | | | | |
|  | **Overall survival**  (n = 1070) | | **Brain cancer specific survival**  (n = 1070) | | **Recurrence**  (n = 1070) | |
| **Predictor** | *Unadjusted HR*  *(95% CI)* | *p-value* | *Unadjusted HR*  *(95% CI)* | *p-value* | *Unadjusted HR*  *(95% CI)* | *p-value* |
| Tumour size (cm) | 1.12  (1.08 – 1.17) | **<0.001*** | 1.19  (1.15 – 1.24) | **<0.001*** | 1.06  (1.00 – 1.12) | **0.037*** |

*(OR – Odds ratio, HR – Hazard ratio, CI – Confidence Interval, SE – Standard Error,* ***** *Statistically significant)*

SA Table 3: Association between tumour size and outcomes of interest (unadjusted) – GBM + Grade 4 subgroup

|  | **Outcome** | | | | | |
| --- | --- | --- | --- | --- | --- | --- |
|  | **30-day mortality**  (n = 510) | | **New or worsened neurological deficit**  (n = 510) | | **Inpatient bed days up to 12 months following diagnosis** (n = 482) | |
| **Predictor** | *Unadjusted OR*  *(95% CI)* | *p-value* | *Unadjusted OR*  *(95% CI)* | *p-value* | *Unadjusted Coefficient (SE)* | *p-value* |
| Tumour size (cm) | 1.54  (1.14 – 2.11) | **0.005*** | 1.02  (0.91 – 1.15) | 0.734 | 1.56 (0.90) | 0.084 |
| **Cont.** | | | | | | |
|  | **Overall survival**  (n = 510) | | **Brain cancer specific survival**  (n = 510) | | **Recurrence**  (n = 510) | |
| **Predictor** | *Unadjusted HR*  *(95% CI)* | *p-value* | *Unadjusted HR*  *(95% CI)* | *p-value* | *Unadjusted HR*  *(95% CI)* | *p-value* |
| Tumour size (cm) | 1.10  (1.04 – 1.16) | **<0.001*** | 1.10  (1.04 – 1.16) | **<0.001*** | 0.89  (0.82 – 0.96) | **0.002*** |

*(OR – Odds ratio, HR – Hazard ratio, CI – Confidence Interval, SE – Standard Error,* ***** *Statistically significant)*

SA Table 4: Association between tumour size and outcomes of interest (unadjusted) Grade 1-3 PBM subgroup

|  | **Outcome** | | | | | |
| --- | --- | --- | --- | --- | --- | --- |
|  | **30-day mortality**  (n = 416) | | **New or worsened neurological deficit**  (n = 416) | | **Inpatient bed days up to 12 months following diagnosis** (n = 334) | |
| **Predictor** | *Unadjusted OR*  *(95% CI)* | *p-value* | *Unadjusted OR*  *(95% CI)* | *p-value* | *Unadjusted Coefficient (SE)* | *p-value* |
| Tumour size (cm) | 0.94  (0.61 – 1.38) | 0.756 | 1.28  (1.11 – 1.48) | **0.001*** | 2.14 (1.13) | 0.059 |
| **Cont.** | | | | | | |
|  | **Overall survival**  (n = 416) | | **Brain cancer specific survival**  (n = 416) | | **Recurrence**  (n = 416) | |
| **Predictor** | *Unadjusted HR*  *(95% CI)* | *p-value* | *Unadjusted HR*  *(95% CI)* | *p-value* | *Unadjusted HR*  *(95% CI)* | *p-value* |
| Tumour size (cm) | 1.15  (1.06 – 1.25) | **0.001*** | 1.24  (1.12 – 1.38) | **<0.001*** | 1.27  (1.15 – 1.41) | **<0.001*** |

*(OR – Odds ratio, HR – Hazard ratio, CI – Confidence Interval, SE – Standard Error,* ***** *Statistically significant)*

SA Table 5: Association between tumour size and outcomes of interest (unadjusted) – Metastatic subgroup

|  | **Outcome** | | | | | |
| --- | --- | --- | --- | --- | --- | --- |
|  | **30-day mortality**  (n = 144) | | **New or worsened neurological deficit**  (n = 144) | | **Inpatient bed days up to 12 months following diagnosis** (n = 133) | |
| **Predictor** | *Unadjusted OR*  *(95% CI)* | *p-value* | *Unadjusted OR*  *(95% CI)* | *p-value* | *Unadjusted Coefficient (SE)* | *p-value* |
| Tumour size (cm) | 1.52  (0.89 – 2.58) | 0.115 | 0.97  (0.64 – 1.43) | 0.867 | 2.15 (2.32) | 0.357 |
| **Cont.** | | | | | | |
|  | **Overall survival**  (n = 144) | | **Brain cancer specific survival**  (n = 144) | | **Recurrence**  (n = 144) | |
| **Predictor** | *Unadjusted HR*  *(95% CI)* | *p-value* | *Unadjusted HR*  *(95% CI)* | *p-value* | *Unadjusted HR*  *(95% CI)* | *p-value* |
| Tumour size (cm) | 1.22  (1.04 – 1.42) | **0.012*** | 1.14  (0.95 – 1.36) | 0.160 | 1.03  (0.82 – 1.30) | 0.771 |

*(OR – Odds ratio, HR – Hazard ratio, CI – Confidence Interval, SE – Standard Error,* ***** *Statistically significant)*

## 5. Time-to-treatment associations with patient outcomes (continuous specification, unadjusted and adjusted)

SA Table 6: Association between the time-to-treatment window and outcomes of interest (unadjusted), Pooled

|  | **Outcome** | | | | | |
| --- | --- | --- | --- | --- | --- | --- |
|  | **Overall survival** | | **Brain cancer-specific survival** | | **Recurrence** | |
| **Predictor** | *Unadjusted HR*  *(95% CI)* | *p-value* | *Unadjusted HR*  *(95% CI)* | *p-value* | *Unadjusted HR (95% CI)* | *p-value* |
| Time radiological diagnosis to first treatment (days) | 0.9963  (0.9952 – 0.9974) | **<0.001*** | 0.9967  (0.9956 – 0.9979) | **<0.001*** | 0.9992  (0.9987 – 0.9998) | **0.006*** |
| Time from first presentation to treatment (days) | 0.9976  (0.9970 – 0.9982) | **<0.001*** | 0.9979  (0.9973 – 0.9985) | **<0.001*** | 0.9993  (0.9989 – 0.9997) | **0.001*** |
| **Cont.** | | | | | | |
|  | **New or worsened neurological deficit** | | | **30-day mortality** | | |
| **Predictor** | *Unadjusted OR (95% CI)* | | *p-value* | *Unadjusted OR (95% CI)* | | *p-value* |
| Time radiological diagnosis to first treatment (days) | 1.000  (1.000 – 1.001) | | 0.718 | 0.930  (0.745 – 0.980) | | 0.380 |
| Time from first presentation to treatment (days) | 1.000  (0.999 – 1.000) | | 0.613 | 0.884  (0.728 – 0.990) | | 0.110 |

SA Table 7: Association between the time-to-treatment window and outcomes of interest (adjusted), Pooled

|  | **Outcome** | | | | | |
| --- | --- | --- | --- | --- | --- | --- |
|  | **Overall survival** | | **Brain cancer-specific survival** | | **Recurrence** | |
| **Predictor** | *Adjusted HR (95% CI)* | *p-value* | *Adjusted HR (95% CI)* | *p-value* | *Adjusted HR (95% CI)* | *p-value* |
| Time radiological diagnosis to first treatment (days) | 0.9979  (0.9971 – 0.9988) | **<0.001*** | 0.9987  (0.9979 – 0.9995) | **0.002*** | 0.9995  (0.9990 – 1.0000) | **0.050*** |
| Time from first presentation to treatment (days) | 0.9985  (0.9980 – 0.9991) | **<0.001*** | 0.9991  (0.9985 – 0.9996) | **0.001*** | 0.9995  (0.9991 – 0.9999) | **0.014*** |
| **Cont.** | | | | | | |
|  | **New or worsened neurological deficit** | | | **30-day mortality** | | |
| **Predictor** | *Adjusted OR (95% CI)* | | *p-value* | *Adjusted OR (95% CI)* | | *p-value* |
| Time radiological diagnosis to first treatment (days) | 1.000  (1.000 – 1.001) | | 0.221 | 0.933  (0.741 – 0.986) | | 0.431 |
| Time from first presentation to treatment (days) | 1.000  (1.000 – 1.001) | | 0.627 | 0.881  (0.689 – 0.975) | | 0.150 |

*(OR – Odds ratio, HR – Hazard ratio, CI – Confidence Interval,* ***** *Statistically significant)*

SA Table 8: Association between the time-to-treatment window and outcomes of interest (unadjusted), GBM + Grade 4

|  | **Outcome** | | | | | |
| --- | --- | --- | --- | --- | --- | --- |
|  | **Overall survival** | | **Brain cancer-specific survival** | | **Recurrence** | |
| **Predictor** | *Unadjusted HR*  *(95% CI)* | *p-value* | *Unadjusted HR*  *(95% CI)* | *p-value* | *Unadjusted HR (95% CI)* | *p-value* |
| Time radiological diagnosis to first treatment (days) | 0.9992  (0.9978 – 1.0006) | 0.243 | 0.9992  (0.9979 – 1.0006) | 0.286 | 0.9994  (0.9976 – 1.0012) | 0.490 |
| Time from first presentation to treatment (days) | 0.9998  (0.9991 – 1.0005) | 0.622 | 0.9997  (0.9989 – 1.0004) | 0.366 | 0.9995  (0.9983 – 1.0006) | 0.364 |
| **Cont.** | | | | | | |
|  | **New or worsened neurological deficit** | | | **30-day mortality** | | |
| **Predictor** | *Unadjusted OR (95% CI)* | | *p-value* | *Unadjusted OR (95% CI)* | | *p-value* |
| Time radiological diagnosis to first treatment (days) | 0.9998  (0.9964 – 1.0024) | | 0.891 | 0.9262  (0.6399 – 1.0104) | | 0.555 |
| Time from first presentation to treatment (days) | 0.9994  (0.9973 – 1.0009) | | 0.501 | 0.8683  (0.6177 – 0.9939) | | 0.257 |

*(OR – Odds ratio, HR – Hazard ratio, CI – Confidence Interval,* ***** *Statistically significant)*

SA Table 9: Association between the time-to-treatment window and outcomes of interest (adjusted), GBM + Grade 4

|  | **Outcome** | | | | | |
| --- | --- | --- | --- | --- | --- | --- |
|  | **Overall survival** | | **Brain cancer-specific survival** | | **Recurrence** | |
| **Predictor** | *Adjusted HR (95% CI)* | *p-value* | *Adjusted HR (95% CI)* | *p-value* | *Adjusted HR (95% CI)* | *p-value* |
| Time radiological diagnosis to first treatment (days) | 0.9989  (0.9973 – 1.0004) | 0.150 | 0.9990  (0.9975 – 1.0005) | 0.210 | 0.9994  (0.9977 – 1.0011) | 0.508 |
| Time from first presentation to treatment (days) | 0.9998  (0.9991 – 1.0005) | 0.557 | 0.9996  (0.9989 – 1.0004) | 0.343 | 0.9996  (0.9985 – 1.0007) | 0.457 |
| **Cont.** | | | | | | |
|  | **New or worsened neurological deficit** | | | **30-day mortality** | | |
| **Predictor** | *Adjusted OR (95% CI)* | | *p-value* | *Adjusted OR (95% CI)* | | *p-value* |
| Time radiological diagnosis to first treatment (days) | 0.9997  (0.9964 – 1.0023) | | 0.823 | 0.9211  (0.6501 – 1.0143) | | 0.511 |
| Time from first presentation to treatment (days) | 0.9994  (0.9973 – 1.0009) | | 0.467 | 0.8671  (0.5168 – 1.0029) | | 0.305 |

*(OR – Odds ratio, HR – Hazard ratio, CI – Confidence Interval,* ***** *Statistically significant)*

SA Table 10: Association between the time-to-treatment window and outcomes of interest (unadjusted), Grade 1-3 PBT

|  | **Outcome** | | | | | |
| --- | --- | --- | --- | --- | --- | --- |
|  | **Overall survival** | | **Brain cancer-specific survival** | | **Recurrence** | |
| **Predictor** | *Unadjusted HR*  *(95% CI)* | *p-value* | *Unadjusted HR*  *(95% CI)* | *p-value* | *Unadjusted HR (95% CI)* | *p-value* |
| Time radiological diagnosis to first treatment (days) | 0.9983  (0.9972 – 0.9994) | **0.003*** | 0.9992  (0.9983 – 1.0001) | 0.065 | 0.9997  (0.9992 – 1.0002) | 0.189 |
| Time from first presentation to treatment (days) | 0.9987  (0.9979 – 0.9994) | **<0.001*** | 0.9994  (0.9988 – 1.0000) | 0.063 | 0.9997  (0.9993 – 1.0001) | 0.131 |
| **Cont.** | | | | | | |
|  | **New or worsened neurological deficit** | | | **30-day mortality** | | |
| **Predictor** | *Unadjusted OR (95% CI)* | | *p-value* | *Unadjusted OR (95% CI)* | | *p-value* |
| Time radiological diagnosis to first treatment (days) | 1.0004  (0.9998 – 1.0009) | | 0.195 | 1.000  (0.000 – 2.272 x 10^40^) | | 1.000 |
| Time from first presentation to treatment (days) | 1.0002  (0.9996 – 1.0006) | | 0.552 | 1.000  (0.000 – 1.140 x 10^35^) | | 1.000 |

*OR – Odds ratio, HR – Hazard ratio, CI – Confidence Interval,* ***** *Statistically significant)*

SA Table 11: Association between the time-to-treatment window and outcomes of interest (adjusted), Grade 1-3 PBT

|  | **Outcome** | | | | | |
| --- | --- | --- | --- | --- | --- | --- |
|  | **Overall survival** | | **Brain cancer-specific survival** | | **Recurrence** | |
| **Predictor** | *Adjusted HR (95% CI)* | *p-value* | *Adjusted HR (95% CI)* | *p-value* | *Adjusted HR (95% CI)* | *p-value* |
| Time radiological diagnosis to first treatment (days) | 0.9984  (0.9973 – 0.9995) | **0.004*** | 0.9992  (0.9983 – 1.0001) | 0.086 | 0.9997  (0.9992 – 1.0001) | 0.161 |
| Time from first presentation to treatment (days) | 0.9987  (0.9980 – 0.9995) | **0.001*** | 0.9995  (0.9988 – 1.0001) | 0.100 | 0.9996  (0.9992 – 1.0000) | 0.079 |
| **Cont.** | | | | | | |
|  | **New or worsened neurological deficit** | | | **30-day mortality** | | |
| **Predictor** | *Adjusted OR (95% CI)* | | *p-value* | *Adjusted OR (95% CI)* | | *p-value* |
| Time radiological diagnosis to first treatment (days) | 1.0003  (0.9997 – 1.0009) | | 0.242 | 1.000  (0.000 – 3.656 x 10^40^) | | 1.000 |
| Time from first presentation to treatment (days) | 1.0001  (0.9996 – 1.0006) | | 0.609 | 1.000  (0.000 – 3.075 x 10^35^) | | 1.000 |

*(OR – Odds ratio, HR – Hazard ratio, CI – Confidence Interval,* ***** *Statistically significant)*

SA Table 12: Association between the time-to-treatment window and outcomes of interest (unadjusted), Metastatic tumours

|  | **Outcome** | | | | | |
| --- | --- | --- | --- | --- | --- | --- |
|  | **Overall survival** | | **Brain cancer-specific survival** | | **Recurrence** | |
| **Predictor** | *Unadjusted HR*  *(95% CI)* | *p-value* | *Unadjusted HR*  *(95% CI)* | *p-value* | *Unadjusted HR (95% CI)* | *p-value* |
| Time radiological diagnosis to first treatment (days) | 0.9995  (0.9979 – 1.0010) | 0.474 | 0.9976  (0.9917 – 1.0034) | 0.411 | 0.9965  (0.9882 – 1.0048) | 0.404 |
| Time from first presentation to treatment (days) | 0.9989  (0.9972 – 1.0007) | 0.249 | 0.9960  (0.9904 – 1.0016) | 0.163 | 0.9979  (0.9936 – 1.0021) | 0.324 |
| **Cont.** | | | | | | |
|  | **New or worsened neurological deficit** | | | **30-day mortality** | | |
| **Predictor** | *Unadjusted OR (95% CI)* | | *p-value* | *Unadjusted OR (95% CI)* | | *p-value* |
| Time radiological diagnosis to first treatment (days) | 0.999  (0.984 – 1.003) | | 0.698 | 0.924  (0.672 – 0.996) | | 0.488 |
| Time from first presentation to treatment (days) | 1.000  (0.993 – 1.003) | | 0.895 | 0.820  (0.485 – 0.977) | | 0.224 |

SA Table 13: Association between the time-to-treatment window and outcomes of interest (adjusted), Metastatic tumours

|  | **Outcome** | | | | | |
| --- | --- | --- | --- | --- | --- | --- |
|  | **Overall survival** | | **Brain cancer-specific survival** | | **Recurrence** | |
| **Predictor** | *Adjusted HR (95% CI)* | *p-value* | *Adjusted HR (95% CI)* | *p-value* | *Adjusted HR (95% CI)* | *p-value* |
| Time radiological diagnosis to first treatment (days) | 0.9998  (0.9982 – 1.0013) | 0.775 | 0.9995  (0.9947 – 1.0043) | 0.831 | 0.9955  (0.9855 – 1.0056) | 0.383 |
| Time from first presentation to treatment (days) | 0.9993  (0.9975 – 1.0012) | 0.475 | 0.9974  (0.9921 – 1.0027) | 0.330 | 0.9978  (0.9933 – 1.0023) | 0.335 |
| **Cont.** | | | | | | |
|  | **New or worsened neurological deficit** | | | **30-day mortality** | | |
| **Predictor** | *Adjusted OR (95% CI)* | | *p-value* | *Adjusted OR (95% CI)* | | *p-value* |
| Time radiological diagnosis to first treatment (days) | 0.998  (0.980 – 1.003) | | 0.632 | 1.015  (0.708 – 1.409) | | 0.917 |
| Time from first presentation to treatment (days) | 0.999  (0.992 – 1.003) | | 0.770 | 0.877  (0.475 – 1.039) | | 0.488 |

*(OR – Odds ratio, HR – Hazard ratio, CI – Confidence Interval,* ***** *Statistically significant)*

## 6. Time-to-treatment associations with patient outcomes, stratified by time-to-treatment category (continuous specification, unadjusted and adjusted)

SA Table 14: Association between the time-to-treatment window (weeks) and outcomes of interest (unadjusted) – Time-to-treatment ≤ 2 weeks

|  | **Outcome** | | | | | |
| --- | --- | --- | --- | --- | --- | --- |
|  | **Overall survival** | | **Brain cancer-specific survival** | | **Recurrence** | |
| **Predictor** | *Unadjusted HR*  *(95% CI)* | *p-value* | *Unadjusted HR*  *(95% CI)* | *p-value* | *Unadjusted HR*  *(95% CI)* | *p-value* |
| Time radiological diagnosis to first treatment (weeks) | 1.1472  (0.9186 – 1.4327) | 0.226 | 1.1411  (0.9010 – 1.4451) | 0.273 | 0.8877  (0.6839 – 1.1522) | 0.371 |
| Time from first presentation to treatment (weeks) | 1.1133  (0.6504 – 1.9058) | 0.695 | 1.2108  (0.6784 – 2.1610) | 0.518 | 0.7607  (0.4040 – 1.4324) | 0.397 |
| **Cont.** | | | | | | |
|  | **New or worsened neurological deficit** | | | **30-day mortality** | | |
| **Predictor** | *Unadjusted OR*  *(95% CI)* | | *p-value* | *Unadjusted OR*  *(95% CI)* | | *p-value* |
| Time radiological diagnosis to first treatment (weeks) | 1.6205  (1.0487 – 2.5406) | | **0.032*** | 4.9652  (0.3163 – 564.2627) | | 0.348 |
| Time from first presentation to treatment (weeks) | 1.4306  (0.4559 – 5.1605) | | 0.556 | 11.9550  (0.1026 – 553972.4311) | | 0.479 |

SA Table 15: Association between the time-to-treatment window (weeks) and outcomes of interest (adjusted) – Time-to-treatment ≤ 2 weeks

|  | **Outcome** | | | | | |
| --- | --- | --- | --- | --- | --- | --- |
|  | **Overall survival** | | **Brain cancer-specific survival** | | **Recurrence** | |
| **Predictor** | *Adjusted HR*  *(95% CI)* | *p-value* | *Adjusted HR*  *(95% CI)* | *p-value* | *Adjusted HR*  *(95% CI)* | *p-value* |
| Time radiological diagnosis to first treatment (weeks) | 1.0148  (0.8032 – 1.2821) | 0.902 | 0.9727  (0.7571 – 1.2498) | 0.829 | 0.8152  (0.6249 – 1.0634) | 0.132 |
| Time from first presentation to treatment (weeks) | 0.9685  (0.5013 – 1.8712) | 0.924 | 1.1344  (0.5669 – 2.2702) | 0.722 | 0.6666  (0.3019 – 1.4720) | 0.316 |
| **Cont.** | | | | | | |
|  | **New or worsened neurological deficit** | | | **30-day mortality** | | |
| **Predictor** | *Adjusted OR*  *(95% CI)* | | *p-value* | *Adjusted OR*  *(95% CI)* | | *p-value* |
| Time radiological diagnosis to first treatment (weeks) | 1.5156  (0.9620 – 2.4199) | | 0.076 | 5.7011  (0.3247 – 822.4359) | | 0.325 |
| Time from first presentation to treatment (weeks) | 0.8835  (0.1892 – 4.2664) | | 0.873 | 3.765 x 10^-23^  (0.0000 – Inf) | | 0.999 |

SA Table 16: Association between the time-to-treatment window (weeks) and outcomes of interest (unadjusted) – Time-to-treatment 2-6 weeks

|  | **Outcome** | | | | | |
| --- | --- | --- | --- | --- | --- | --- |
|  | **Overall survival** | | **Brain cancer-specific survival** | | **Recurrence** | |
| **Predictor** | *Unadjusted HR*  *(95% CI)* | *p-value* | *Unadjusted HR*  *(95% CI)* | *p-value* | *Unadjusted HR*  *(95% CI)* | *p-value* |
| Time radiological diagnosis to first treatment (weeks) | 0.9194  (0.8247 – 1.0251) | 0.130 | 0.8871  (0.7914 – 0.9943) | **0.040*** | 0.8580  (0.7399 – 0.9950) | **0.043*** |
| Time from first presentation to treatment (weeks) | 1.0110  (0.8974 – 1.1389) | 0.857 | 0.9857  (0.8666 – 1.1211) | 0.826 | 0.8803  (0.7570 – 1.0237) | 0.098 |
| **Cont.** | | | | | | |
|  | **New or worsened neurological deficit** | | | **30-day mortality** | | |
| **Predictor** | *Unadjusted OR*  *(95% CI)* | | *p-value* | *Unadjusted OR*  *(95% CI)* | | *p-value* |
| Time radiological diagnosis to first treatment (weeks) | 0.9642  (0.7738 – 1.1959) | | 0.742 | 1.0000  (0.0000 – Inf) | | 1.000 |
| Time from first presentation to treatment (weeks) | 0.9743  (0.7688 – 1.2345) | | 0.829 | 0.0000  (NA – Inf) | | 0.994 |

SA Table 17: Association between the time-to-treatment window (weeks) and outcomes of interest (adjusted) – Time-to-treatment 2-6 weeks

|  | **Outcome** | | | | | |
| --- | --- | --- | --- | --- | --- | --- |
|  | **Overall survival** | | **Brain cancer-specific survival** | | **Recurrence** | |
| **Predictor** | *Adjusted HR*  *(95% CI)* | *p-value* | *Adjusted HR*  *(95% CI)* | *p-value* | *Adjusted HR*  *(95% CI)* | *p-value* |
| Time radiological diagnosis to first treatment (weeks) | 0.9829  (0.8726 – 1.1070) | 0.776 | 0.9829  (0.8674 – 1.1137) | 0.787 | 0.8647  (0.7426 – 1.0069) | 0.061 |
| Time from first presentation to treatment (weeks) | 1.0739  (0.9535 – 1.2096) | 0.240 | 1.0275  (0.9066 – 1.1644) | 0.671 | 0.8915  (0.7631 – 1.0416) | 0.148 |
| **Cont.** | | | | | | |
|  | **New or worsened neurological deficit** | | | **30-day mortality** | | |
| **Predictor** | *Adjusted OR*  *(95% CI)* | | *p-value* | *Adjusted OR*  *(95% CI)* | | *p-value* |
| Time radiological diagnosis to first treatment (weeks) | 1.0112  (0.8042 – 1.2677) | | 0.923 | 1.0000  (0.0000 – Inf) | | 1.000 |
| Time from first presentation to treatment (weeks) | 0.9965  (0.7810 – 1.2720) | | 0.978 | 0.0000  (0.0000 – Inf) | | 0.996 |

SA Table 18: Association between the time-to-treatment window (weeks) and outcomes of interest (unadjusted) – Time-to-treatment 6-12 weeks

|  | **Outcome** | | | | | |
| --- | --- | --- | --- | --- | --- | --- |
|  | **Overall survival** | | **Brain cancer-specific survival** | | **Recurrence** | |
| **Predictor** | *Unadjusted HR*  *(95% CI)* | *p-value* | *Unadjusted HR*  *(95% CI)* | *p-value* | *Unadjusted HR*  *(95% CI)* | *p-value* |
| Time radiological diagnosis to first treatment (weeks) | 0.7479  (0.6224 – 0.8987) | **0.002*** | 0.7613  (0.6199 – 0.9351) | **0.009*** | 0.9941  (0.8296 – 1.1912) | 0.949 |
| Time from first presentation to treatment (weeks) | 0.8993  (0.8138 – 0.9939) | **0.037*** | 0.9360  (0.8424 – 1.0401) | 0.219 | 0.9059  (0.8012 – 1.0243) | 0.115 |
| **Cont.** | | | | | | |
|  | **New or worsened neurological deficit** | | | **30-day mortality** | | |
| **Predictor** | *Unadjusted OR*  *(95% CI)* | | *p-value* | *Unadjusted OR*  *(95% CI)* | | *p-value* |
| Time radiological diagnosis to first treatment (weeks) | 1.0164  (0.7559 – 1.3489) | | 0.911 | 1.0000  (0.0000 – Inf) | | 1.000 |
| Time from first presentation to treatment (weeks) | 0.8661  (0.7044 – 1.0560) | | 0.162 | 1.0000  (0.0000 – Inf) | | 1.000 |

SA Table 19: Association between the time-to-treatment window (weeks) and outcomes of interest (adjusted) – Time-to-treatment 6-12 weeks

|  | **Outcome** | | | | | |
| --- | --- | --- | --- | --- | --- | --- |
|  | **Overall survival** | | **Brain cancer-specific survival** | | **Recurrence** | |
| **Predictor** | *Adjusted HR*  *(95% CI)* | *p-value* | *Adjusted HR*  *(95% CI)* | *p-value* | *Adjusted HR*  *(95% CI)* | *p-value* |
| Time radiological diagnosis to first treatment (weeks) | 0.8360  (0.6755 – 1.0346) | 0.099 | 0.8172  (0.6439 – 1.0370) | 0.097 | 1.1189  (0.9054 – 1.3828) | 0.298 |
| Time from first presentation to treatment (weeks) | 0.9818  (0.8825 – 1.0922) | 0.735 | 1.0227  (0.9137 – 1.1447) | 0.696 | 0.9135  (0.8048 – 1.0369) | 0.162 |
| **Cont.** | | | | | | |
|  | **New or worsened neurological deficit** | | | **30-day mortality** | | |
| **Predictor** | *Adjusted OR*  *(95% CI)* | | *p-value* | *Adjusted OR*  *(95% CI)* | | *p-value* |
| Time radiological diagnosis to first treatment (weeks) | 1.0498  (0.7511 – 1.4617) | | 0.772 | 1.0000  (0.0000 – Inf) | | 1.000 |
| Time from first presentation to treatment (weeks) | 0.8578  (0.6875 – 1.0595) | | 0.162 | 1.0000  (0.0000 – Inf) | | 1.000 |

SA Table 20: Association between the time-to-treatment window (weeks) and outcomes of interest (unadjusted) – Time-to-treatment > 12 weeks

|  | **Outcome** | | | | | |
| --- | --- | --- | --- | --- | --- | --- |
|  | **Overall survival** | | **Brain cancer-specific survival** | | **Recurrence** | |
| **Predictor** | *Unadjusted HR*  *(95% CI)* | *p-value* | *Unadjusted HR*  *(95% CI)* | *p-value* | *Unadjusted HR*  *(95% CI)* | *p-value* |
| Time radiological diagnosis to first treatment (weeks) | 0.9942  (0.9889 – 0.9996) | **0.035*** | 0.9946  (0.9889 – 1.0003) | 0.062 | 0.9962  (0.9921 – 1.0003) | 0.066 |
| Time from first presentation to treatment (weeks) | 0.9895  (0.9850 – 0.9940) | **<0.001*** | 0.9912  (0.9867 – 0.9957) | **<0.001*** | 0.9966  (0.9935 – 0.9997) | **0.030*** |
| **Cont.** | | | | | | |
|  | **New or worsened neurological deficit** | | | **30-day mortality** | | |
| **Predictor** | *Unadjusted OR*  *(95% CI)* | | *p-value* | *Unadjusted OR*  *(95% CI)* | | *p-value* |
| Time radiological diagnosis to first treatment (weeks) | 1.0039  (0.9995 – 1.0084) | | 0.075 | 1.0000  (0.0000 – Inf) | | 1.000 |
| Time from first presentation to treatment (weeks) | 1.0007  (0.9967 – 1.0042) | | 0.717 | 1.0000  (0.0000 – ~Inf) | | 1.000 |

SA Table 21: Association between the time-to-treatment window (weeks) and outcomes of interest (adjusted) – Time-to-treatment > 12 weeks

|  | **Outcome** | | | | | |
| --- | --- | --- | --- | --- | --- | --- |
|  | **Overall survival** | | **Brain cancer-specific survival** | | **Recurrence** | |
| **Predictor** | *Adjusted HR*  *(95% CI)* | *p-value* | *Adjusted HR*  *(95% CI)* | *p-value* | *Adjusted HR*  *(95% CI)* | *p-value* |
| Time radiological diagnosis to first treatment (weeks) | 0.9940  (0.9886 – 0.9994) | **0.029*** | 0.9950  (0.9895 – 1.0005) | 0.073 | 0.9963  (0.9923 – 1.0004) | 0.075 |
| Time from first presentation to treatment (weeks) | 0.9940  (0.9900 – 0.9979) | **0.003*** | 0.9962  (0.9922 – 1.0001) | 0.057 | 0.9971  (0.9940 – 1.0001) | 0.059 |
| **Cont.** | | | | | | |
|  | **New or worsened neurological deficit** | | | **30-day mortality** | | |
| **Predictor** | *Adjusted OR*  *(95% CI)* | | *p-value* | *Adjusted OR*  *(95% CI)* | | *p-value* |
| Time radiological diagnosis to first treatment (weeks) | 1.0043  (0.9997 – 1.0089) | | 0.059 | 1.0000  (0.0000 – Inf) | | 1.000 |
| Time from first presentation to treatment (weeks) | 1.0016  (0.9976 – 1.0054) | | 0.407 | 1.0000  (0.0000 – Inf) | | 1.000 |

## 7. Number of Observations in Regression Analysis

Note the number of observations included in each analysis varies depending on the necessary variables and levels of missingness.

SA Table 22: Number of Observations, Tumour size associations with patient outcomes

|  | **All patients** | | **GBM and Grade 4 non-GBM** | | **Grade 1 to 3 non-GBM** | | **Metastasis** | |
| --- | --- | --- | --- | --- | --- | --- | --- | --- |
|  | Unadj. | Adj. | Unadj. | Adj. | Unadj. | Adj. | Unadj. | Adj. |
| All-cause mortality  Brain cancer mortality  Recurrence  30-day mortality  Neuro deficit | 1070 | 1064 | 510 | 508 | 416 | 413 | 144 | 143 |
| Inpatient bed days (12 months following diag.) | 949 | 944 | 482 | 480 | 334 | 332 | 133 | 132 |

SA Table 23: Number of Observations, Time-to-treatment associations with patient outcomes (Diagnosis to treatment

|  | **All patients** | | **GBM and Grade 4 non-GBM** | | **Grade 1 to 3 non-GBM** | | **Metastasis** | |
| --- | --- | --- | --- | --- | --- | --- | --- | --- |
|  | Unadj. | Adj. | Unadj. | Adj. | Unadj. | Adj. | Unadj. | Adj. |
| All-cause mortality  Brain cancer mortality  Recurrence  30-day mortality  Neuro deficit | 911 | 905 | 446 | 444 | 362 | 359 | 103 | 102 |

SA Table 24: Number of Observations, Time-to-treatment associations with patient outcomes - (First presentation to treatment)

| **Time-to-treatment associations with patient outcomes - (First presentation to treatment**) | **All patients** | | **GBM and Grade 4 non-GBM** | | **Grade 1 to 3 non-GBM** | | **Metastasis** | |
| --- | --- | --- | --- | --- | --- | --- | --- | --- |
|  | Unadj. | Adj. | Unadj. | Adj. | Unadj. | Adj. | Unadj. | Adj. |
| All-cause mortality  Brain cancer mortality  Recurrence  30-day mortality  Neuro deficit | 871 | 866 | 434 | 432 | 340 | 338 | 97 | 96 |

## 8.Supplementary Figures

Figure SA1 - Forest Plot with Subgroups, Main Outcomes
